# Interpretation of Cardiac and Non-Cardiac Causes of Elevated Troponin T Levels in Non-Acute Coronary Syndrome Patients in the Emergency Department

**DOI:** 10.7759/cureus.22703

**Published:** 2022-02-28

**Authors:** Hany A Zaki, Ahmed E Shaban, Amira E Shaban, Eman E Shaban

**Affiliations:** 1 Emergency Medicine, Hamad Medical Corporation, Doha, QAT; 2 Internal Medicine, Mansoura General Hospital, Mansoura, EGY; 3 Medicine, Mansoura University, Mansoura, EGY; 4 Internal Medicine, Mansoura University Hospital, Mansoura, EGY; 5 Cardiology, Al Jufairi Diagnosis And Treatment, Doha, QAT

**Keywords:** high troponin t, systemic inflammatory response syndrome, septic shock, acute pericarditis, pulmonary embolism (pe), chronic heart failure, strenuous exercise, cardiac troponin i, cardiac enzymes

## Abstract

The definition of myocardial infarction was updated in 2000 to include an elevation of cardiac troponin T or I (cTnT or xTnI) alongside clinical evidence of myocardial infarction. The redefinition was jointly done by the American College of Cardiology Committee and the European Society of Cardiology. Since then, cardiac troponin T and I have assumed the position as the primary biochemical markers for diagnosing myocardial infarction. The high sensitivity of cardiac troponin for myocardial necrosis influenced the decision to include cardiac troponins (cTn) in the diagnostic pathway. An elevated cTn level indicates the presence of myocardial injury. However, it does not give the underlying reason for the damage. Apart from acute myocardial infarction (AMI), a range of potential diseases feature troponin release, including heart failure, acute pulmonary embolism, end-stage renal disease, and myocarditis. However, regardless of the mechanism that triggers the release from cardiac myocytes, elevated cTnI and cTnT typically implies a poor prognosis. This review attempts to explain both the cardiac and non-cardiac causes of increased cTnT in emergency department patients.

## Introduction and background

**Figure 1:**
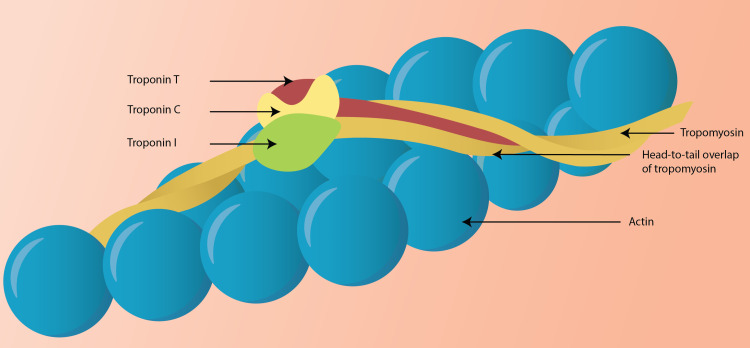
Morphology of the troponin complex in cardiac muscle Troponin is a three-unit complex of troponin T, I, and C (T for ‘tropomyosin-binding,’ I for ‘inhibitory,’ and C for ‘calcium-binding’). Troponin I (TnI) and T (TnT) have cardiac-specific isoforms and are used for assessing the cardiac injury

Cardiac troponin T (cTnT), cardiac troponin I isoforms (cTnI), and cardiac troponin C (cTnC) are typically expressed in the cardiac muscle tissues (Figure 1).

It is important to note that they are the most sensitive and most specific indicators for acute myocardial infarction (AMI) diagnosis [1-4]. Not many studies have reported the cTnT and cTnI expression outside the myocardium, especially in the skeletal muscle [5-7]. However, these studies all have contradictory results and have not been verified in other research studies [8, 9].

The techniques for determining cTnT and cTnI were developed in the late 90s [10, 11] and have undergone improvement, enhancing analytical specificity and sensitivity. This helped speed up the early diagnosis of AMI and drastically minimize the number of false-positive results due to anti-cTnT and anti-cTnI antibody interaction with the release of skeletal troponins from muscle fibers in skeletal myopathies and rhabdomyolysis [12-14]. Currently, there are a lot of sensitive immunoassays that help determine the concentrations of high-sensitive (hs)-cTnT and hs-cTnI with the capacity to detect low concentrations of cardiac troponin in blood serum [15, 16]. The concentration of cardiac troponin, though small, is diagnostically significant. This has accelerated AMI diagnosis significantly, thus enhancing decision-making on therapy for patients presenting with chest pain. For example, usage of hs-cTnI and hs-cTnT in three-hour and one-hour diagnostic algorithms are regulated by the Fourth Universal Definition of Myocardial Infarction (2018) [1].

It is important to note that despite the specificity of cTnI and cTnT, one cannot classify them as ideal biomarkers for AMI diagnosis because their concentrations increase in blood serum both in ischemic necrosis of myocardial cells and also in several other pathologies that lead to damage or death of myocardial cells due to non-ischemic factors [17-19]. It is also worth mentioning that improved analytical sensitivity of techniques for cardiac troponin determination has reduced specificity - a condition expressed by frequent cases of increased troponin levels without myocardial infarction [15]. On the other hand, it is clinically relevant due to the opportunity to assess better the extent of myocardial damage in several pathological conditions, including pulmonary embolism (PE), sepsis, and inflammatory myocardial ailments. Still, conversely, it can increase the difficulty of differential diagnosis and contribute to wrong diagnoses. This is true in situations where clinicians heavily rely on the results of laboratory examinations.

It should also be noted that an increase in cardiac troponin concentrate in biological fluids, mainly in the blood serum, is an indication of reversible or irreversible damage to the cardiomyocytes but fails to explain the etiopathogenesis of the damage, which may occur in several other pathological processes not linked to myocardial infarction [1, 14, 20]. In addition, in some instances (false-positive results, impaired renal filtration), cardiac troponins may be elevated even with no cardiomyocyte damage [12, 13]. As such, researchers and practitioners should not discard any data on the possibility of cardiac troponin increase in other medical conditions.

Elucidation of primary causes and mechanisms of cardiac troponin increase that is of theoretical value and practical importance [21]. This plays a vital role in improving the differential diagnosis of myocardial infarction from other medical conditions that occur alongside non-ischemic alteration of cardiomyocytes.

This article will discuss the primary causes and the mechanisms of increased cardiac troponin T concentration in emergency department patients.

## Review

Cardiac troponin release, unrelated to acute coronary syndrome

Septic Shock/Sepsis and Systemic Inflammatory Response Syndrome

According to results from several studies, over 36% of patients treated in the emergency department for systemic inflammatory response syndrome (SIRS) or sepsis have elevated cTn. The range is more comprehensive, i.e., 36% to 85% of cases [22]. This wide range of prevalence is because sepsis has different causes. It is also due to the different troponin assays and the different cut-off values applied. In most cases, coronary artery disease has been excluded, indicating that there are other underlying mechanisms to these troponin elevations. A key factor for cTn release from damaged myocardial cells may be a mismatch between oxygen demand-supply the oxygen demand of the myocardium increases due to tachycardia and fever. Simultaneously, there is a shortfall in the supply of oxygen to the myocardium due to systemic hypoxemia arising from respiratory failure, hypotension, anemia in some cases, and microcirculatory dysfunction. Also, local and circulating inflammatory markers such as interleukin 6, tumor necrosis factor α, reactive oxygen species, plus bacterial endotoxins may cause direct injury to the myocardium by cytotoxic effects. It is also important to note that high cTn values are a source of prognostic information, and the extent to which cTn is elevated correlates with the disease process. Ver Elst et al. showed a strong association between cTnI-positivity and left ventricular (LV)-dysfunction (78% vs. 9% in cTnI-negative patients; p < 0.001). there was a significant correlation between the values of cTnI with APACHE II score and the degree of hypotension [23].

Pulmonary Embolism

Pulmonary embolism (PE) is a primary cause of troponin elevation. Over half of patients feel a so-called "coronary pain, " resulting in difficulties and wrong differential diagnosis [24, 25].

A study by Giannitis and colleagues [26] found that the increase in cardiac troponin concentration in PE depends on the volume of the damage done to the pulmonary vascular bed. In severe pulmonary embolism, increased troponin levels were observed in over 50% of patients. It is also important to note that elevated cTnT levels in pulmonary embolism were associated with cardiogenic shock, a high hospital mortality rate, and the need for resuscitation. In addition, patients who had high cTnT levels and PE were more likely to require mechanical ventilation and inotropic support compared with PE patients who were troponin-negative [27].

According to a 2012 study by Kilinc et al. [28], elevated cTnI levels had a higher frequency in patients with PE (50.8% vs. 11.6%; p<0.001) - an indication of a moderate diagnostic value of cardiac troponins in pulmonary embolism [29]. If we are to follow the analogy with the research results of Giannitis et al. [28], the troponin level was dependent on the volume of damage done to the pulmonary vascular bed. For example, at least 80% of patients with severe PE had elevated cTnI levels. Increased cTnI levels were observed in submassive PE cases (56%) and 38% nonmassive PE. The researchers discovered that elevated cTnI levels for PE diagnosis had such analytical parameters as a specificity of 88% sensitivity of 51%. Analysis of the diagnostic efficiency of combined high levels of D-dimer and cTnI showed a sensitivity of 93%. However, the specificity fell to 54% [29]. A meta-analysis of 20 clinical trials and 1985 patients showed that cardiac troponins have a high predictive value in pulmonary embolism. For instance, a survey of 618 patients with increased cardiac troponin levels revealed that 122 died (19.7%; 95% CI).

On the other hand, a survey of 1367 subjects with reference troponin values revealed a lower mortality rate (3.7%; 95% CI). Also, cardiac troponin I and troponin T had almost similar predictive values. A review of the meta-analysis results shows that high levels of cardiac troponins in the serum are associated with short-term mortality in PE patients (5.24 OR; 95% CI). A review of a group of PE patients who were hemodynamically stable found that elevated serum troponin concentrations were associated with an increased risk of mortality (5.90 OR; 95% CI) [30]. And so, by focusing on the high cardiac troponin concentrations in PE, a clinician can identify patients who have an increased risk of complications or poor prognosis. Many notable researchers and experts have concluded that the predictive value of cardiac troponins is high in PE, as shown in clinical guidelines. It is important to note that cardiac troponins are recommended for predicting and stratification of risk in PE patients [31, 32].

Acute and Chronic Heart Failure

Elevated cardiac troponin in heart failure strongly links with decreased left ventricular ejection fraction. Elevated cTn also correlates with the severity of heart failure (HF) and the prognosis. The aggravation of ischemic or non-ischemic heart failure is attributed to the progressive loss of myocytes caused by apoptosis and necrosis. Other factors, such as the activation of the renin-angiotensin-aldosterone system and the sympathetic nervous system and inflammatory mediators, may also trigger an injury to the myocardium. Myocytes lost are replaced by fibrotic tissue resulting in progressive cardiac dysfunction. Elevation of cTn in heart failure patients indicates myocardial injury. In decompensated heart failure, cTn release is believed to be caused by excessive tension from the myocardium wall due to acute volume and pressure overload. Also, increased strain on the myocardial wall results in subendocardial ischemia. In patients with chronic stable heart failure, high cTnI values occurred in 15-23% of cases (> 0.1 ng/ml). In the case of cTnT, values exceeding 0.1 ng/ml have been reported in at least 10-15% of cases [33]. No difference was found between the ischemic and non-ischemic groups [33]. Over 52-55% of the acute HF patients admitted to the facility had increased cTnT values. When cTn occurs in HF, it indicates a poor short and long-term outcome. Patients whose troponin values are on the high side have lower ejection fractions, high clinical grading of heart failure, and higher mortality [34]. It is also important to note that serial cTn measurements can provide prognostic information. A decrease in cTn concentration is associated with improved left ventricular function. On the other hand, persistently increased troponin values were readily expressed in patients who eventually died.

Strenuous Exercise

Results from several studies have reported the appearance of cTnI and cTnT after strenuous ultra-endurance exercise [35]. But then, we do not have a complete understanding of elevated cardiac troponin mechanisms, nor do we have a sense of its prognostic significance. However, after prolonged strength training, only transient increases of small amounts of cTn normalized or decreased within 24 hours after the activity was detected [35]. In addition, the plasma changes differ significantly from those found in myocardial infarction (MI). This birthed the assumption that an increase in cTn level could result from a transient release of cTnI and cTnT and not from the constant release of troponin after myocardial necrosis. Conversely, autopsy results and extensive cardiac examinations have shown significant distortion of the heart structure and acute coronary heart disease in athletes who were presumed to be healthy. As such, the elevation of cTn levels could be triggered by an underlying subclinical heart disease unmasked by strength training.

Acute Pericarditis

Acute pericarditis/myocarditis is typically diagnosed in patients in the emergency department presenting with acute chest pain. While troponins may not be present in the pericardium, it was reported that cTnI was elevated in 32-49% of pericarditis cases due to the involvement of the epicardium in the process of inflammation. In patients presenting with acute myocarditis, cTnI concentrations are seen to increase in no less than 34% of patients [36].

Coronary angiography is usually performed in patients who have pericarditis. The aim is to rule out myocardial infarction. In the absence of coronary disease, endomyocardial biopsies (EMB) are done to establish an accurate diagnosis. However, EMB shows lymphocytic and myocytolysis infiltrates in only 10 - 25% of patients with myocarditis. Increased cTnT values have a higher sensitivity than EMB and may have the capacity to confirm the clinical diagnosis of myocarditis [36].

Chronic Renal Failure

Looking at modern concepts, one may consider cardiac troponins to be products of normal myocardial metabolism [37], and there's a lack of troponin-negative patients. These concepts were birthed due to the establishment of the most recent immunoassays for the detection of cardiac troponin [38, 39]. For example, cardiac troponin molecules can be detected in all healthy patients using ultrasensitive test systems. The concentration of the molecules is usually below the threshold level. On the other hand, if one exceeds this threshold, the concentrations of troponin will be considered abnormal, and clinicians should review the possible causes of elevation.

Because of the presence of cardiac troponins in healthy patients, researchers are now compelled to consider what causes their release from cardiomyocytes under normal circumstances [37]. It is also worth mentioning that the mechanism by which troponin molecules are eliminated from the general circulation is a major factor that may affect the concentration of troponin in the serum. Currently, troponins are eliminated from the blood serum through the following methods:

a - In reticuloendothelial system cells (intracellular cleavage by certain proteases)

b - In the serum (extracellular cleavage by proteolytic enzymes). It has been established that thrombin (an enzyme of the hemostasis system) causes the troponin T molecule to cleave into two fragments.

c - Glomerular filtration

Glomerular filtration, without a doubt, is the most discussed and possibly the primary mechanism by which cardiac troponin is eliminated with regards to clinical significance. Some researchers have reported that the involvement of the kidneys in the elimination of cardiac troponin is highly doubtful and controversial, owing to the fact that cardiac troponin molecules were not detected in the urine of the majority of the patients [40]. Regardless, high concentrations of cardiac troponin in renal patients without any symptoms of cardiovascular disease have been seen several times in clinical practice [36]. These findings are an indication that the renal system is involved in the elimination of serum troponin.

## Conclusions

Based on this review, it can be concluded that several factors can contribute to the elevation of cardiac troponin concentration. The pathological conditions (chronic renal failure, pulmonary embolism, sepsis, and inflammatory myocardial disease) and the physiological conditions (strength training) are very common causes of high cardiac troponin concentration. It is also important to note that modern sensitive techniques for the determination of cardiac troponin detect troponin molecules at a higher frequency and in a larger number of individuals at the same time. It is worth mentioning that, for the sake of increasing a key criterion of lab diagnostics, the developers gave up another key criterion for diagnostics (specificity of immunoassays). Clinicians should consider this circumstance when interpreting laboratory results of patients admitted with possible acute myocardial infarction. Otherwise, the chances of misdiagnosis in the blood serum after these are numerous and differ from the mechanism of increased cardiac troponins in acute MI. Taking note of the mechanisms of high-sensitivity cardiac troponin concentration elevation will improve the differential diagnosis of acute myocardial infarction from the conditions listed above and also introduce additional diagnostic techniques. For instance, one can determine high-sensitivity troponins in serum after pharmacological or physical exercise, thus troponins can serve as markers of latent ischemic disease.
